# A lesson in homology

**DOI:** 10.7554/eLife.48335

**Published:** 2019-06-18

**Authors:** Nikola-Michael Prpic

**Affiliations:** Allgemeine Zoologie und EntwicklungsbiologieJustus-Liebig-Universität GießenGießenGermany

**Keywords:** cephalopod, cuttlefish, homology, limb development, limb evolution, evodevo, Other

## Abstract

The same genes and signaling pathways control the formation of limbs in vertebrates, arthropods and cuttlefish.

**Related research article** Tarazona OA, Lopez DH, Slota LA, Cohn MJ. 2019. Evolution of limb development in cephalopod mollusks. *eLife* **8**:e43828. doi: 10.7554/eLife.43828

A human leg appears to have little in common with an insect leg, apart from the fact that both are used for walking, so most zoologists think that the limbs of vertebrates and arthropods evolved independently and are not, therefore, homologous structures. Ask a developmental geneticist, however, and the answer will not be so clear. Research in the fly *Drosophila melanogaster* has identified a number of genes and signaling pathways that guide the development of the legs along the three axes of the body: anterior-posterior (head-to-tail), dorsal-ventral (top-to-bottom), and proximal-distal, which runs from the body to the tips of the limbs (reviewed in Estella et al., 2012).

In flies, two proteins, Extradenticle and Homothorax, are co-expressed in the part of the leg closest to the body and they establish an initial proximal-distal axis in the developing limb. The distal part is further refined by ‘leg-gap genes’, which are triggered by molecular signals active in this area, such as the Wnt, Hedgehog and Bmp signaling pathways. Two of these pathways, Wnt and Hedgehog, are also involved in setting up the anterior-posterior axis of the leg; another two, Wnt and Bmp, collaborate to form the dorsal-ventral orientation.

Subsequent research revealed that an almost identical set of genes and signaling pathways control the development of limbs in chicken and mouse (reviewed in Pueyo and Couso, 2005). Is this just a coincidence, or a hint that zoologists should reconsider their take on the evolutionary history of vertebrate and arthropod limbs?

The truth may actually lie somewhere in between. According to the ‘co-option hypothesis’, a developmental program evolved in the common ancestor of the bilaterians – a group that includes most animals except for primitive forms like sponges – to shape an appendage that later disappeared during evolution. However, the program itself survived in arthropods, vertebrates and possibly other bilaterians, where it would have been independently repurposed to build limbs (Gaunt, 1997; Shubin et al., 1997; Tabin et al., 1999; Pueyo and Couso, 2005). As such, the appendage program would be homologous, but the structures that it helps to shape would not.

In 2005, Pueyo and Couso proposed a way to test the co-option hypothesis: "if conservation of similar features are found in the tentacles of a cephalopod [...], then conservation of an ancestral appendage developmental program cannot be ignored" (Pueyo and Couso, 2005). Now, in eLife, Oscar Tarazona, Davys Lopez, Leslie Slota and Martin Cohn of the University of Florida report that more than a dozen genes in the conserved appendage program are also expressed in the developing arms and tentacles of two cephalopod mollusks, the cuttlefishes *Sepia officinalis* and *Sepia bandensis* (Tarazona et al., 2019).

Just like arthropod and vertebrate limbs, cuttlefish appendages have a proximal part that co-expresses *Extradenticle* and *Homothorax*, and a distal part that expresses homologs of the leg-gap genes as well as components of the Wnt, Hedgehog, and Bmp signaling pathways. In addition, the expression patterns of the genes closely resemble those in arthropod and vertebrate limbs.

These results alone are intriguing, but Tarazona et al. took the analysis one step further, examining how Bmp and Hedgehog signaling helped to form cephalopod limbs. Tiny beads soaked with a chemical that inhibits Bmp signaling were implanted on the dorsal side of the arms and tentacles, which led to suckers abnormally appearing in this area. These results show that, as in arthropods and vertebrates, Bmp signaling is required for proper dorsal-ventral development in cephalopods.

The team also tested the role of Hedgehog signaling. Tissue from donor embryos that expressed Hedgehog was transplanted into developing cuttlefish, which generated a second anterior-posterior axis in the arms and tentacles. On the other hand, repressing the pathway with the drug cyclopamine dramatically reduced the axis in the limbs. These stunning results leave no doubt that the segmental legs of arthropods, the limbs of vertebrates, and the arms or tentacles of cephalopods use very similar developmental genetic mechanisms. While this does not indicate that these limbs are homologous, the findings strongly support the co-option hypothesis.

To rephrase the conclusions by Tarazona et al., the ancestral appendage program is probably not a ‘Sleeping Beauty’ which lies dormant in the genome of limbless creatures, waiting to be repurposed once in a while. Instead, it was always patterning some kind of appendage, implying that limbless animals evolved from animals with limbs. Extinct members of the major branches of bilaterians all appear to have some sort of frontal extensions (Figure 1). Did the appendage program initially evolve to build these structures? The answer to this question might come from finding more exceptionally preserved fossils, and from carefully sampling the role of the appendage program in other present-day animals.

**Figure 1.**
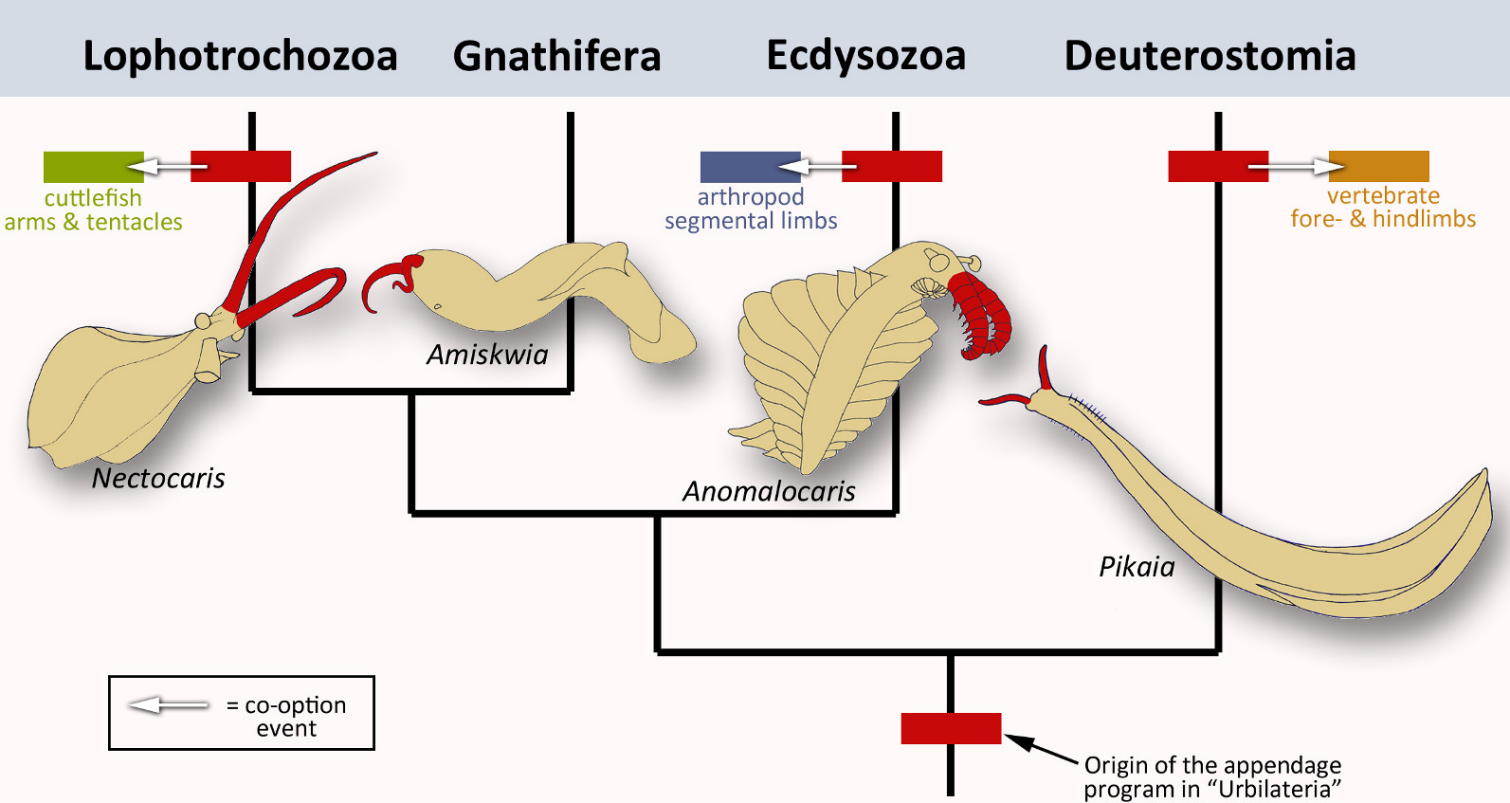
The evolution of animal limbs. Bilaterian animals have both an anterior-posterior and dorsal-ventral axis: they encompass lophotrochozoans (e.g. cuttlefish), gnathiferans, ecdysozoans (e.g. arthropods) and deuterostomes (e.g. vertebrates). Tarazona et al. imply that an appendage program originated in the common ancestor, urbilateria (red box at bottom of the bilaterian phylogenetic tree; after Marlétaz et al., 2019). This program would still exist in the lophotrochozoans, ecdysozoans and deuterostomes – the situation in the gnathiferans has not been studied yet. In each group, the program has been co-opted to create new appendages such as vertebrate limbs, arthropod legs or cephalopod tentacles and arms (arrows and corresponding colored boxes). These lineages are strongly divergent, yet the appendage program is conserved in each of them. This prompted Tarazona et al. to conclude that the program has remained active during evolution. Interestingly, pairs of frontal appendages (shown in red) have been found in fossils belonging to all four branches. Did the appendage program initially evolve to build these structures? Image credit: *Nectocaris:* after Smith and Caron, 2010; *Amiskwia:* after Caron and Cheung, 2019; *Anomalocaris:* after Collins, 1996; *Pikaia:* after Morris and Caron, 2012.
